# Predicting social media post popularity using multimodal deep learning: Insights from content features

**DOI:** 10.1371/journal.pone.0356612

**Published:** 2026-08-26

**Authors:** Yi Wang, Aoyu Zeng

**Affiliations:** School of Business, Central University of Finance and Economics, Beijing, China; Rutgers The State University of New Jersey, UNITED STATES OF AMERICA

## Abstract

This study predicts social media post popularity by examining the associations between multimodal, unstructured content features and observed engagement outcomes and by developing scalable, data-driven tools for social media content assessment. Grounded in Diffusion of Innovations, we conceptualize post popularity and the mechanisms of user engagement, extract visual and textual features from real-world social media data, validate their relationships with popularity, and build two deep learning models—one estimating numerical popularity and one predicting popularity levels. Results show that multimodal features, including visual and textual attributes, are significantly associated with popularity, and both models demonstrate useful predictive performance within the evaluated data setting. We extend the concept of post popularity by proposing a staged classification framework that groups posts into four levels based on aggregated engagement outcomes and demonstrate how multimodal feature analysis coupled with deep learning–based prediction offers new theoretical insight into content dissemination while yielding scalable tools for optimization. Practically, our approach enables marketers to assess popularity at both quantitative and categorical levels, supporting informed content planning, targeted promotion, and resource allocation, and helping brands move from intuition-driven to data-driven strategies.

## 1 Introduction

Driven by digitalization, social media has transcended traditional communication models, becoming a core domain for brand value creation and reshaping brand-consumer interactions [1]. By the end of 2024, the number of active social media users worldwide reached 5.17 billion, accounting for over 63% of the global population. On average, users spend 2 hours and 24 minutes daily on social media, with 73.8% of internet users regularly searching for brand-related information [2]. These figures highlight social media as both a space for real-time interaction and a powerful channel for brand exposure and market competitiveness.

Highly popular posts stand out for their ability to quickly attract user attention through likes, shares, and comments, significantly boosting brand exposure and engagement. User-generated content (UGC) from these posts helps reduce marketing costs and provides timely feedback, enabling brands to refine their offerings [3,4]. This drives sales and enhances brand value [5,6].

Although post popularity appears uncertain, prior research suggests that it is shaped by identifiable content and contextual factors [1,7–9]. However, existing studies often examine text or image features separately and rely heavily on structured metadata. Less is known about how multimodal content features can be integrated to predict different levels of post popularity. This gap limits brands’ ability to identify high-potential content before publication.

To address this, our study develops a theoretical framework to understand user engagement and post popularity. Drawing on Diffusion of Innovations Theory [10] and multimodal content research [1,11,12], we conceptualize post popularity as a staged outcome based on different levels of engagement. We then examine how image aesthetics, image content, and textual features relate to popularity and develop a multimodal deep learning model to predict popularity outcomes.

We analyze posts from four verified brand accounts on Sina Weibo, extracting visual (e.g., hue, saturation, facial presence), textual (e.g., sentiment, hashtags, mentions), and metadata (e.g., post time, follower count) features. These features are validated using regression analysis and random forest–based feature importance ranking. Based on these, we create two models: (1) a continuous prediction model estimating specific engagement values, and (2) a categorical model predicting popularity levels (low, moderate, high, viral).

This study makes three contributions. First, it extends post popularity from a single engagement outcome to a staged classification framework. Second, it identifies how visual, textual, temporal, and account-level features relate to post popularity. Third, it develops and evaluates a multimodal prediction model that combines VGG19-based image representations with structured content features.

## 2 Literature review

In this section, we first define the staged categories of post popularity, then explain the user engagement mechanisms driving popularity evolution. We further review prior studies on multimodal feature influences and conclude by positioning our study’s contribution.

### 2.1 *The formation of post popularity and user engagement*

Social media content dissemination often follows a staged and cumulative trajectory shaped by network structure, user behavior, and content appeal, rather than a simple linear process. Prior research typically defines post popularity as the degree of user engagement—measured by likes, comments, and reposts [13–15]. Building on this view, we conceptualize popularity as a staged outcome. Drawing on the Diffusion of Innovations Theory [10], we borrow its notion of sequential adoption but adapt it to classify posts by levels of engagement intensity, rather than by user groups. This adaptation allows us to capture the “long-tail” distribution patterns of social media interactions, consistent with prior findings on online content diffusion [7,9].

Accordingly, we classify post popularity into four levels:

- ***Low-popularity posts***: posts with minimal interaction and limited visibility within the network;
- ***Moderate-popularity posts***: posts attracting steady, routine interaction and reach without exceptional amplification;
- ***High-popularity posts***: posts achieving strong recognition and broad visibility beyond the core audience;
- ***Viral posts*:** posts diffusing rapidly across audiences and/or platforms and generating outsized impact.

**Fig 1:**
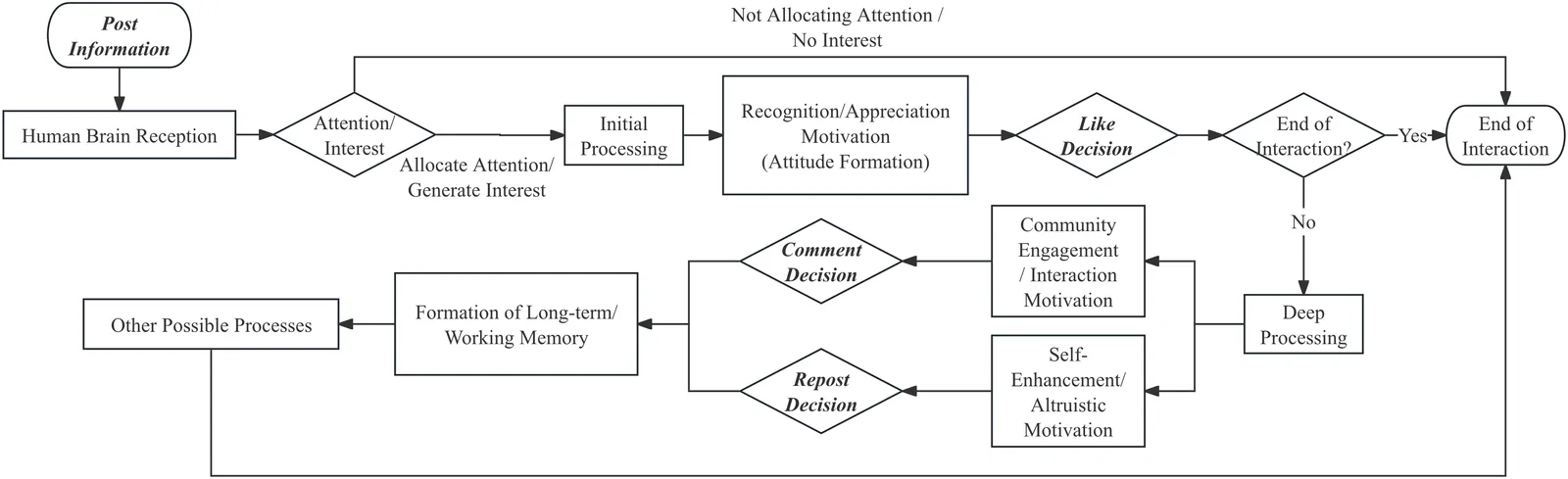
Theoretical model of the mechanism underlying user engagement behavior.

Beyond structural classification, post popularity is also driven by cognitive and motivational mechanisms of user engagement. Likes fulfill emotional needs, comments provide cognitive feedback or interaction, and reposts express identity or altruism [16–18]. Information processing theory suggests engagement progresses from perceptual filtering to deeper processing when stimuli such as novelty, relevance, or emotional appeal capture attention [19]. Intrinsic motivations—community involvement, personal growth, value sharing—further propel the shift from passive exposure to active interaction [20]. For example, users are more likely to like posts with positive emotional tones [16], comment on content that sparks reflection [21], and repost when the content aligns with identity or altruistic goals [19]. Fig 1 illustrates how post information is filtered through attention and initial processing before generating different forms of user engagement. Likes represent an initial recognition or appreciation response, whereas comments and reposts are associated with deeper processing and motivations related to community interaction, self-enhancement, and altruistic sharing.

### 2.2 *Post image features and post popularity*

Images influence post popularity by shaping attention, processing fluency, and emotional response [22]. Visual aesthetics and image content can make posts more salient, easier to process, and more likely to trigger user engagement [1].

#### 2.2.1 *Aesthetic features of post images and post popularity.*

Composition and visual balance guide viewers’ attention and reduce cognitive load, making image information easier to process [23,24]. Foreground-background contrast increases subject salience and supports efficient visual communication, consistent with Gestalt principles [25,26]. Color attributes such as hue, saturation, brightness, clarity, and contrast further affect attention and affective response: warm colors and high saturation can enhance attention and emotional arousal, while low clarity or excessive contrast may increase cognitive load [27–29].

#### 2.2.2 *Content features of post images and post popularity.*

Image content features such as faces and brand logos can also affect engagement. Faces can shape consumers’ affective and informational responses to online visual content [30]. In social media settings, consumer-selfie images featuring faces have also been associated with higher levels of likes and comments [7]. However, they may also distract attention from product or brand information [30]. Similarly, brand logos serve as visual signals that support recognition and emotional connection [31,32], but overly prominent logos may heighten persuasion awareness and reduce engagement [31].

### 2.3 *Textual features of posts and post popularity*

Textual features may influence post popularity through information richness, interaction cues, and emotional framing.

#### 2.3.1 *Fundamental textual features of posts and post popularity.*

Text length serves as a fundamental metric for assessing the informational richness of posts and has a significant impact on user engagement and post popularity. Studies have shown that longer texts often contain more information, effectively capturing user attention and fostering interaction. However, excessively long texts may increase cognitive load, thereby reducing users’ willingness to engage and diminishing post popularity [33,34].

#### 2.3.2 *Interactive textual features of posts and post popularity.*

Interactive features within posts play a crucial role in driving user engagement and enhancing post popularity. Studies have shown that the use of interactive elements (such as hashtags “#” and mention tags “@”) significantly increases post visibility, thereby attracting greater user attention and engagement [6]. Furthermore, the strategic use of interactive components not only expands the reach of information, enabling sustained exposure across a broader audience, but also effectively stimulates user interactions (such as likes, comments, and reposts), thereby positively influencing post popularity [35].

#### 2.3.3 *Content text features of post and post popularity.*

Emotional tone, brand mentions, and other linguistic features in post text play a crucial role in shaping user engagement and enhancing post popularity. Studies have shown that posts containing positive emotional elements or explicit brand cues can evoke users’ emotional resonance and cognitive responses, thereby significantly increasing user engagement [12]. These textual features not only enhance the appeal of information but also effectively drive user interactions, providing strong support for the formation of post popularity [36].

### 2.4 *Research on predicting social media post popularity*

Prior research has examined the drivers of online content dissemination and virality. Emotional and contextual factors such as humor, timeliness, unexpectedness, and emotional intensity can increase dissemination effectiveness and user interaction [37,38], while user behaviors such as posting and commenting can create social support networks that accelerate information flow [16,19,20]. Other studies have developed models to detect viral potential by combining content, temporal, social, and diffusion-related features [39–41].

However, many existing approaches rely on statistical or shallow machine learning models and often emphasize structured metadata rather than rich multimodal content. As a result, they may have limited ability to capture nonlinear relationships, high-level visual semantics, and cross-modal interactions between text and images. This study addresses this gap by developing a multimodal deep learning framework for post popularity prediction.

### 2.5 *Research framework*

To address these gaps, our study adopts a two-stage approach. First, we analyze the relationships between multimodal features—visual aesthetics, image content, and textual semantics—and post popularity. By identifying significant features, we establish a theory- and data-driven foundation for model development. Based on this, we propose a deep learning–based multimodal prediction framework.

**Fig 2:**
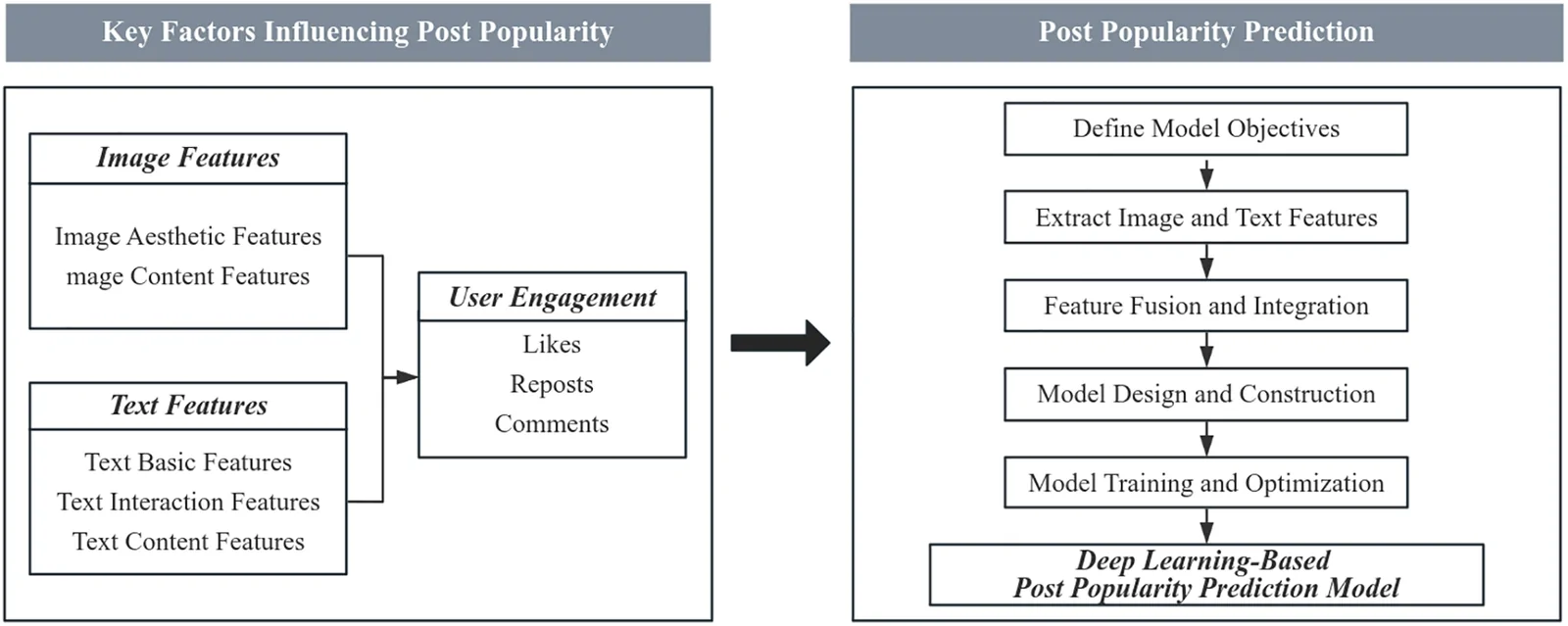
Research framework.

Using computer vision and natural language processing techniques, we construct two models: (1) a continuous regression model that estimates post popularity based on engagement, and (2) a categorical model that predicts popularity levels (low, moderate, high, viral). While we don’t model diffusion pathways directly, these perspectives help identify content signals with high popularity potential. Fig 2 summarizes the two-stage research framework. The first stage identifies visual and textual features associated with likes, comments, and reposts, while the second stage integrates these multimodal features into a deep learning framework for model construction, training, and post popularity prediction.

## 3 Research design

This section describes the research design in four steps: data collection, variable measurement, explanatory analysis, and prediction model construction.

### 3.1 *Sample and data sources*

The data were collected from Sina Weibo, a major social media platform in China. The study focuses on firm-generated content posted by verified official brand accounts, including post text, images, posting time, likes, comments, and reposts. Only publicly available post-level information was collected, and no private messages, restricted profiles, or non-public personal information were accessed. The data were used solely for academic research in accordance with the platform’s terms and conditions at the time of collection.

The sample includes official accounts of Baidu, BMW, Lenovo, and China Mobile, representing the internet services, automobile, technology, and telecommunications industries, respectively. All four accounts were verified, posted regularly, and had more than 100,000 followers. The dataset covers posts published between February 2011 and October 2015. Accordingly, likes, comments, and reposts represent cumulative engagement observed at the collection date. Days Since Posting was included as a temporal control because it captures both the nominal time available for engagement accumulation and broader differences across publication periods; it should therefore not be interpreted as a pure measure of exposure duration.

Although the data were collected during an earlier stage of social media development, they provide standardized textual, visual, temporal, account-level, and engagement information suitable for evaluating the methodological feasibility of multimodal post popularity prediction. To ensure a clear correspondence between textual and visual content, the main analysis retained only single-image posts with valid text, images, posting time, and engagement information. Posts with missing values, long images, low sentiment-confidence scores, or abnormal image-color values were excluded, resulting in a final sample of 9,807 posts.

### 3.2 *Variables and measurement*

After defining the data sources, the variables involved in our study are described in detail (see Table 1 for variable descriptions). The explanatory variables were selected based on the theoretical mechanisms and prior empirical findings reviewed in Section 2, covering visual, textual factors associated with social media engagement.

**Table 1:** Overview of variables.

Variable Measurement	Specific Indicator	Definition
A. Post Popularity		
User Engagement	Number of Likes	The number of likes received by each post.
	Number of Comments	The number of comments received by each post.
	Number of Reposts	The number of reposts received by each post.
B. Image Features		
Aesthetic Features – Visual Balance	Color Visual Balance	Average Euclidean distance between RGB values of left–right symmetrical pixel pairs w.r.t. the vertical midline; higher score indicates lower color balance.
Aesthetic Features – Subject Separation	Size Difference	Difference in area share between subject and background; larger value = greater size difference.
	Color Difference	Euclidean distance between mean RGB of subject and background; larger value = greater color contrast.
Aesthetic Features – Color Characteristics	Hue	Mean HSV hue over all pixels.
	Saturation	Mean HSV saturation over all pixels; higher = more vivid colors.
	Value	Mean HSV value/brightness over all pixels; higher = brighter image.
	Definition	Image sharpness measured by the Laplacian focus metric; higher = clearer image.
	Contrast	Local contrast measured as the mean squared deviation of grayscale values from four-neighbor pixels; higher = greater contrast.
Image Content Features	Face Present	1 if a face is detected, otherwise 0.
	Brand Logo Present	1 if a brand logo corresponding to the posting brand is detected, otherwise 0.
C. Text Features		
Fundamental	Word Count	Number of words in the post text.
Interactive	Number of Hashtags	Count of hashtags “#” in the text.
	Number of Mentions	Count of mentions “@” in the text.
Content	Brand Name Mention (in text)	1 if the posting brand’s name is detected in text; otherwise 0.
	Sentiment Orientation	Probability (0–1) that the text is positive (API score).

#### 3.2.1 *Measurement of post popularity.*

Post popularity is operationalized using three observable user engagement indicators: likes, comments, and reposts [14,42,43]. We also construct total engagement as the unweighted sum of these indicators to capture the overall volume of interactions received by a post. Because likes are more frequent than comments and reposts, this aggregate measure represents total interaction volume rather than an equally balanced multidimensional engagement construct. Therefore, likes, comments, and reposts are also analyzed separately.

#### 3.2.2 *Measurement of multimodal content and contextual features.*

Aesthetic features of images are measured across three dimensions: visual balance, subject-background separation, and color characteristics. Color Visual Balance is computed as the average Euclidean distance of RGB values between mirrored pixels (left–right). Higher score indicates greater color imbalance. Subject-background separation includes size and color differences, calculated by the area ratio and the Euclidean distance of RGB values between the subject and background. Color characteristics include hue, saturation, value, definition, and contrast. Hue, saturation, and value represent the average color properties of the image, while definition is measured using the Laplacian operator, and contrast is calculated by the mean squared deviation of grayscale values from neighboring pixels.

Content features include the presence of faces and brand logos in the image, detected using the Baidu AI Cloud APIs. Textual features are divided into three categories: fundamental, interactive, and content features. Fundamental features are based on word count, interactive features include the number of hashtags (#) and mentions (@), and content features include brand name detection and sentiment orientation, determined via Python scripts and sentiment analysis (Baidu AI Cloud API). Additionally, post timestamps and follower count are incorporated as temporal and social influence indicators.

### 3.3 *Model design*

To examine the relationships between multimodal features and post popularity and to develop predictive tools for content assessment, our study employs association analysis and predictive modeling strategies.

#### 3.3.1 *Regression model design.*

To analyze the relationships between content features and post popularity, an OLS regression model was constructed, as represented by the following functional form:


$$\begin{array}{c} \text{Post Popularity}=\beta_{\text{0}}+\beta_{\text{1}}\cdot \text{Img}\_\text{aes}+\beta_{\text{2}}\cdot \text{Img}\_\text{cont}+\beta_{\text{3}}\cdot \text{Text}\_\text{feat}+\gamma \cdot \text{Controls} \end{array}$$ (1)

$$\begin{array}{c} \text{Likes}=\alpha_{\text{0}}+\alpha_{\text{1}}\cdot \text{Img}\_\text{aes}+\alpha_{\text{2}}\cdot \text{Img}\_\text{cont}+\alpha_{\text{3}}\cdot \text{Text}\_\text{feat}+\gamma_{\text{1}}\cdot \text{Controls} \end{array}$$ (1.1)

$$\begin{array}{c} \text{Comments}=\mu_{\text{0}}+\mu_{\text{1}}\cdot \text{Img}\_\text{aes}+\mu_{\text{2}}\cdot \text{Img}\_\text{cont}+\mu_{\text{3}}\cdot \text{Text}\_\text{feat}+\gamma_{\text{2}}\cdot \text{Controls} \end{array}$$ (1.2)

$$\begin{array}{c} \text{Reposts}=\phi_{\text{0}}+\phi_{\text{1}}\cdot \text{Img}\_\text{aes}+\phi_{\text{2}}\cdot \text{Img}\_\text{cont}+\phi_{\text{3}}\cdot \text{Text}\_\text{feat}+\gamma_{\text{3}}\cdot \text{Controls} \end{array}$$ (1.3)

In this model, post popularity is quantified as the sum of likes, comments, and reposts, reflecting the overall engagement level of a post. This aggregate metric has been widely used in prior literature as an indicator of post popularity on social media platforms. The independent variables include vectors for image aesthetic features (Img_aes), image content features (Img_cont), textual features (Text_feat), and control variables. Because comments and reposts include zero values, all engagement outcomes were transformed using ln(1+x), where x denotes the original number of likes, comments, reposts, or total engagement. This transformation retains zero-valued observations while reducing the influence of the highly right-skewed engagement distributions.

However, the dataset does not contain post-level measures of algorithmic exposure, paid amplification, or campaign support. Because these unobserved factors may be correlated with both content characteristics and engagement outcomes, the estimated coefficients should be interpreted as conditional associations rather than unbiased causal effects.

#### 3.3.2 *Prediction model design.*

**Fig 3:**
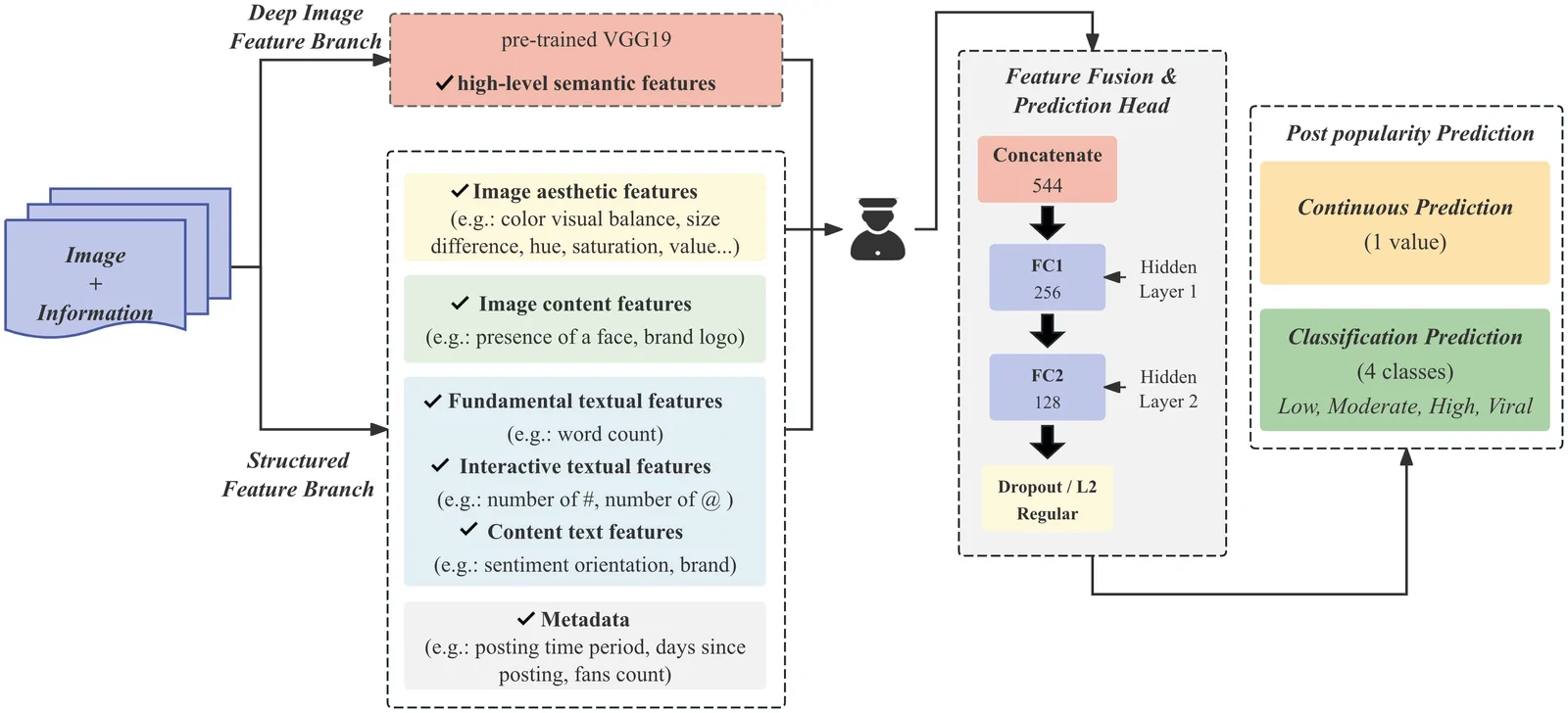
Architecture of the multimodal post popularity prediction model.

To predict post popularity, we develop a multimodal deep learning model that combines two complementary sources of information. One branch extracts latent visual representations from post images using VGG19, while the other processes interpretable visual, textual, temporal, and account-level features. The two representations are fused to support two prediction tasks: continuous engagement estimation and popularity-level classification. Fig 3 provides an overview of the model, while detailed architecture and training settings are reported in Section 5.

## 4 Analysis of multimodal features associated with post popularity

### 4.1 *Descriptive statistics*

This part summarizes key variables related to user engagement and post popularity, laying the empirical groundwork for subsequent modeling and analysis.

#### 4.1.1 *Post popularity on social media platforms.*

Table 2 reports the descriptive statistics for post popularity and related visual, textual, and contextual features. Engagement metrics show substantial variation, especially comments and reposts, which follow long-tail distributions with extreme values. This pattern is consistent with social media content dynamics, where only a small share of posts becomes highly popular while most receive limited engagement. Therefore, the skewed distribution supports our four-level popularity classification based on engagement percentiles.

**Table 2:** Descriptive statistics of variables.

Variable	Min	Max	Mean	SD
A: Post Popularity				
Number of Likes	1	28144	3754.73	3982.10
Number of Comments	0	413525	393.79	8346.78
Number of Reposts	0	1025628	925.52	17445.41
B: Multimodal Content and Contextual Features				
Color Visual Balance	1.74	319.32	85.43	35.34
Size Difference	-95.19	99.04	2.53	45.56
Color Difference	2.62	429.41	176.69	104.57
Hue	0	360	174.69	28.79
Saturation	1.63	251.54	91.82	48.42
Value	4.83	254.35	162.44	56.14
Definition	24.83	72685.31	2484.72	3952.57
Contrast	517.7	29297.6	17986.4	6424.88
Face Present	0	1	0.39	0.49
Brand Logo Present	0	1	0.29	0.45
Word Count	9	165	85.95	48.99
Number of Hashtags	0	4	0.64	0.59
Number of Mentions	0	3	0.22	0.78
Brand Name Mention (in text)	0	1	0.49	0.50
Sentiment Orientation	0	1	0.78	0.35

#### 4.1.2 *Descriptive patterns of visual and textual features.*

Visual inspection of the sample images reveals several patterns. Most images show dispersed color balance and high brightness (value), often skewed toward cooler hues. Foreground-background separation varies, with significant size and color contrast in many cases. Notably, 39% of the images contain human faces, while 29% contain identifiable brand logos.

Textually, the average post contains about 86 words, consistent with the platform’s short-form style. However, interactive elements are underutilized: the average number of hashtags (#) is 0.64, and mentions (@) appear in just 22% of posts. Approximately half include brand names. Sentiment analysis shows an overall positive tone, though negative and neutral posts still make up a notable minority.

These findings suggest that while Weibo brand content shows some visual and textual consistency, there is significant variability in content design, interactivity, and emotional framing. This variation provides a basis for examining the associations between multimodal features and post popularity in the following sections.

### 4.2 *Associations between multimodal features and post popularity*

Our study employs the Ordinary Least Squares (OLS) method to construct a multiple linear regression model, specifying four different models based on variations in the dependent variable. The corresponding regression results are presented in Table 3.

**Table 3:** Multiple linear regression results.

DV	Likes		Comments		Reposts		User Engagement	
IV	Coefficient	P-value	Coefficient	P-value	Coefficient	P-value	Coefficient	P-value
Intercept	2.68***	0.00 (0.07)	3.26***	0.00 (0.09)	3.79***	0.00 (0.12)	4.36***	0.00 (0.10)
Color Visual Balance	−0.03***	0.00 (0.01)	−0.02	0.14 (0.01)	−0.05***	0.01 (0.02)	−0.03	0.05 (0.02)
Size Difference	−0.04	0.22 (0.03)	0.01	0.77 (0.04)	0.06	0.30 (0.06)	0.03	0.55 (0.05)
Color Difference	0.02	0.62 (0.05)	0.08	0.15 (0.06)	0.12	0.14 (0.07)	0.10	0.15 (0.06)
Hue	−0.03***	0.00 (0.01)	0.00	0.87 (0.01)	0.00	0.87 (0.02)	0.00	0.88 (0.01)
Saturation	0.02	0.12 (0.01)	0.10***	0.00 (0.02)	0.14***	0.00 (0.02)	0.11***	0.00 (0.02)
Value	−0.09***	0.00 (0.02)	−0.05*	0.06 (0.02)	−0.06	0.11 (0.03)	−0.05*	0.10 (0.03)
Definition	−0.01	0.49 (0.01)	0.01	0.30 (0.01)	0.04**	0.04 (0.02)	0.02	0.17 (0.01)
Contrast	0.02	0.21 (0.01)	−0.02	0.21 (0.02)	0.00	0.97 (0.02)	0.00	0.89 (0.02)
Face Present	−0.05***	0.01 (0.02)	−0.07***	0.00 (0.03)	0.06*	0.09 (0.03)	−0.01	0.76 (0.03)
Brand Logo Present	0.05**	0.02 (0.02)	0.03	0.32 (0.03)	−0.04	0.22 (0.04)	0.00	0.94 (0.03)
Word Count	−0.03***	0.00 (0.01)	0.05***	0.00 (0.02)	0.21***	0.00 (0.02)	0.11***	0.00 (0.02)
Number of Hashtags	0.13***	0.00 (0.02)	0.39***	0.00 (0.02)	0.60***	0.00 (0.03)	0.46***	0.00 (0.03)
Number of Mentions	0.01	0.39 (0.01)	0.10***	0.00 (0.01)	0.10***	0.00 (0.02)	0.10***	0.00 (0.01)
Brand Name Mention (in text)	0.01	0.80 (0.02)	0.14***	0.00 (0.03)	0.02	0.63 (0.04)	0.08**	0.01 (0.03)
Sentiment Orientation	−0.01	0.35 (0.01)	−0.07***	0.00 (0.02)	−0.05**	0.04 (0.03)	−0.03	0.13 (0.02)
**CV**								
Time Period 2 (10:00–14:00)	−0.17***	0.00 (0.03)	−0.24***	0.00 (0.04)	−0.27***	0.00 (0.05)	−0.23***	0.00 (0.04)
Time Period 3 (14:00–18:00)	−0.27***	0.00 (0.03)	−0.40***	0.00 (0.03)	−0.46***	0.00 (0.04)	−0.40***	0.00 (0.04)
Time Period 4 (18:00–22:00)	−0.12***	0.00 (0.03)	−0.36***	0.00 (0.04)	−0.41***	0.00 (0.06)	−0.35***	0.00 (0.05)
Time Period 5 (22:00–02:00)	−0.09***	0.00 (0.03)	−0.08**	0.05 (0.04)	−0.23***	0.00 (0.05)	−0.167***	0.00 (0.04)
Time Period 6 (02:00–06:00)	0.02	0.54 (0.04)	−0.34***	0.00 (0.05)	−0.34***	0.00 (0.07)	−0.28***	0.00 (0.06)
Days Since Posting	−0.73***	0.00 (0.01)	−0.16***	0.00 (0.01)	−0.04**	0.04 (0.02)	−0.18***	0.00 (0.01)
**Adjusted R²**	0.523		0.164		0.216		0.179	
**Model F-Test**	F = 342.3, P = 0.00		F = 62.09, P = 0.00		F = 86.78, P = 0.00		F = 69.05, P = 0.00	

note: *** p < 0.01, ** p < 0.05, * p < 0.1.

#### 4.2.1 *Associations of image aesthetic features with post popularity.*

Regression analysis shows that the color visual balance metric is significantly negatively correlated with likes (β = −0.03, p = 0.00) and reposts (β = −0.05, p = 0.01). Because higher values indicate greater color imbalance, images with relatively more balanced color distributions tend to receive more likes and reposts [23,24].

In subject-background separation, size and color differences are not significantly associated with the engagement metrics, indicating that these separation measures provide limited explanatory information for engagement in the present sample.

Regarding color, hue is negatively correlated with likes (β = −0.03, p = 0.00), suggesting warmer tones may be more emotionally stimulating than cooler ones [44]. Saturation is positively correlated with comments (β = 0.10, p = 0.00), reposts (β = 0.14, p = 0.00), and overall engagement (β = 0.11, p = 0.00), indicating that posts with more saturated images tend to receive higher levels of engagement [45]. Value is negatively associated with likes (β = −0.09, p = 0.00) and comments (β = −0.05, p = 0.06), suggesting overly bright images reduce contrast and clarity, dampening user interest [29]. Definition is positively associated with reposts (β = 0.04, p = 0.04), likely because clear images are perceived as more professional and shareable [28]. Contrast is not significantly associated with post popularity.

#### 4.2.2 *Associations of image content features with post popularity.*

In image content features, the presence of a face is negatively associated with likes (β = −0.05, p = 0.01) and comments (β = −0.07, p = 0.00), but positively correlated with reposts (β = 0.06, p = 0.09). Faces may divert attention from brand elements or reduce visual clarity, leading to fewer likes and comments [30]. The positive association with reposts may be consistent with greater perceived relatability or expressiveness [7].

In contrast, the presence of a brand logo is positively correlated only with likes (β = 0.05, p = 0.02), suggesting that its predictive relationship may be stronger for lightweight engagement than for more effortful forms of interaction.

#### 4.2.3 *Associations of textual features with post popularity.*

Regression analysis shows that word count is negatively correlated with likes (β = −0.03, p = 0.00) but positively correlated with comments (β = 0.05, p = 0.00), reposts (β = 0.21, p = 0.00), and overall engagement (β = 0.11, p = 0.00). Shorter posts are more likely to get likes, while longer posts provide more information, encouraging comments and shares.

Hashtag count is positively correlated with likes (β = 0.13, p = 0.00), comments (β = 0.39, p = 0.00), reposts (β = 0.60, p = 0.00), and overall engagement (β = 0.46, p = 0.00), suggesting that hashtags improve discoverability and engagement [35]. Mentions (@) are positively correlated with comments (β = 0.10, p = 0.00), reposts (β = 0.10, p = 0.00), and engagement (β = 0.10, p = 0.00), indicating that posts containing more mentions tend to receive more comments and reposts [46].

The presence of a brand name is positively correlated with comments (β = 0.14, p = 0.00) and engagement (β = 0.08, p = 0.01), suggesting that mentioning the brand boosts user attention and discussion [12]. In contrast, positive sentiment is negatively correlated with comments (β = −0.07, p = 0.00) and reposts (β = −0.05, p = 0.04), indicating that emotionally charged or slightly negative content prompts stronger reactions than overly positive posts, which may seem bland or less urgent [11,38].

Days Since Posting is negatively associated with likes (β=−0.73, p = 0.0) and shows smaller negative associations with comments, reposts, and total engagement. This result indicates that more recently published posts tended to receive higher engagement in the observed sample, despite having less nominal time to accumulate interactions. This pattern may reflect changes over time in account reach, platform activity, or user behavior, as social media engagement is often concentrated shortly after publication. Therefore, the coefficient should be interpreted as a broad temporal association rather than as the isolated effect of engagement accumulation time. Although Days Since Posting is an important temporal predictor, several visual and textual features remain significantly associated with engagement after this temporal control is included, indicating that content features provide additional predictive information beyond the observed temporal context.

#### 4.2.4 *Robustness.*

Breusch–Pagan tests indicate heteroskedasticity across the OLS specifications (all p < 0.001); therefore, we report HC3-robust standard errors. VIF diagnostics show high collinearity between the two subject–background separation metrics, so we conduct a robustness check by replacing them with a standardized composite index, SB_index = z(size difference) + z(color difference). After aggregation, all VIFs are below 3 (max = 2.80), suggesting no severe multicollinearity. Additional robustness checks using negative binomial models, GLM–negative binomial models, and quantile regressions provide broad support for the main findings, while also revealing some model-specific and tail-specific heterogeneity. Detailed results are reported in S1 Appendix.

### 4.3 *Predictive importance of multimodal features*

**Fig 4:**
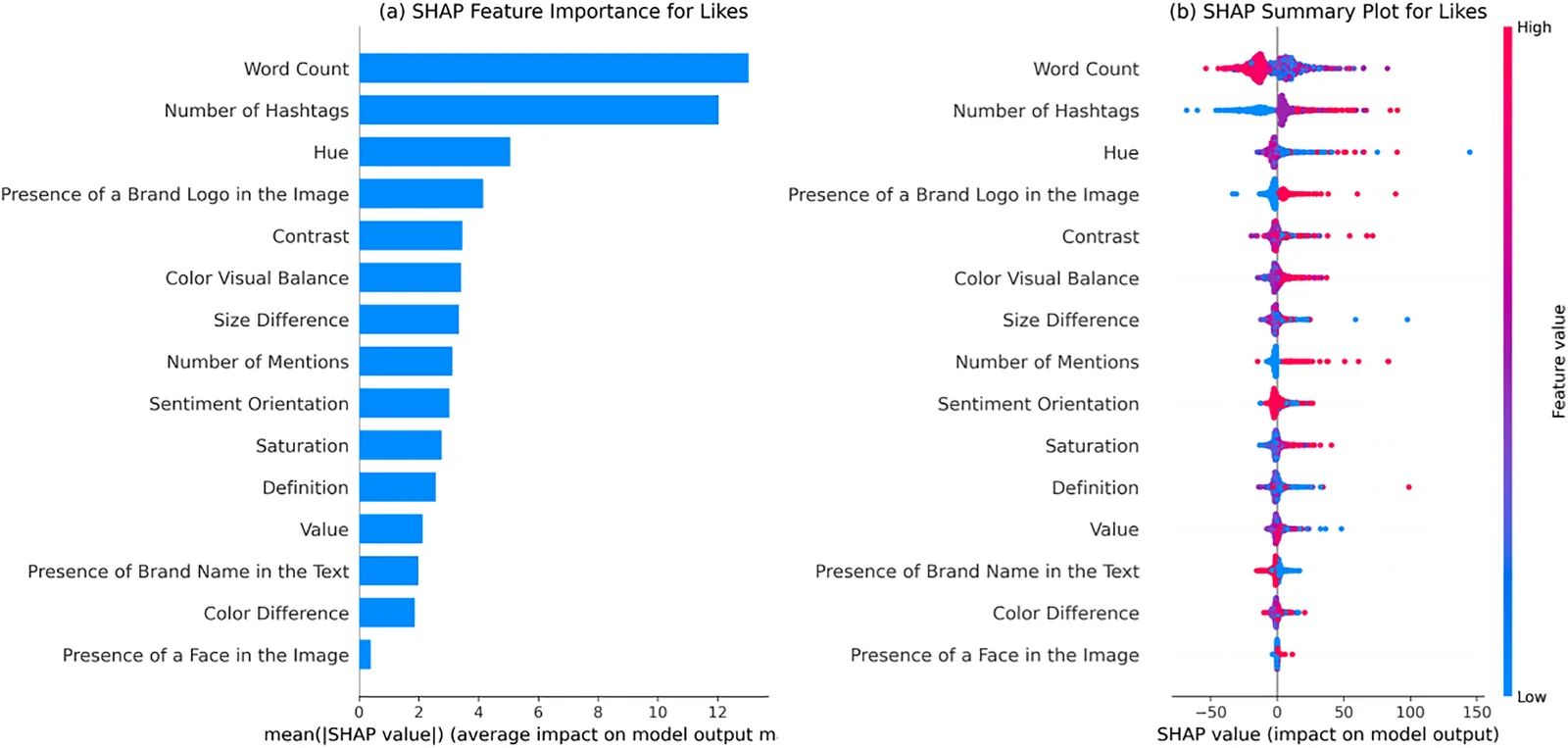
SHAP feature importance for predicting likes.

**Fig 5:**
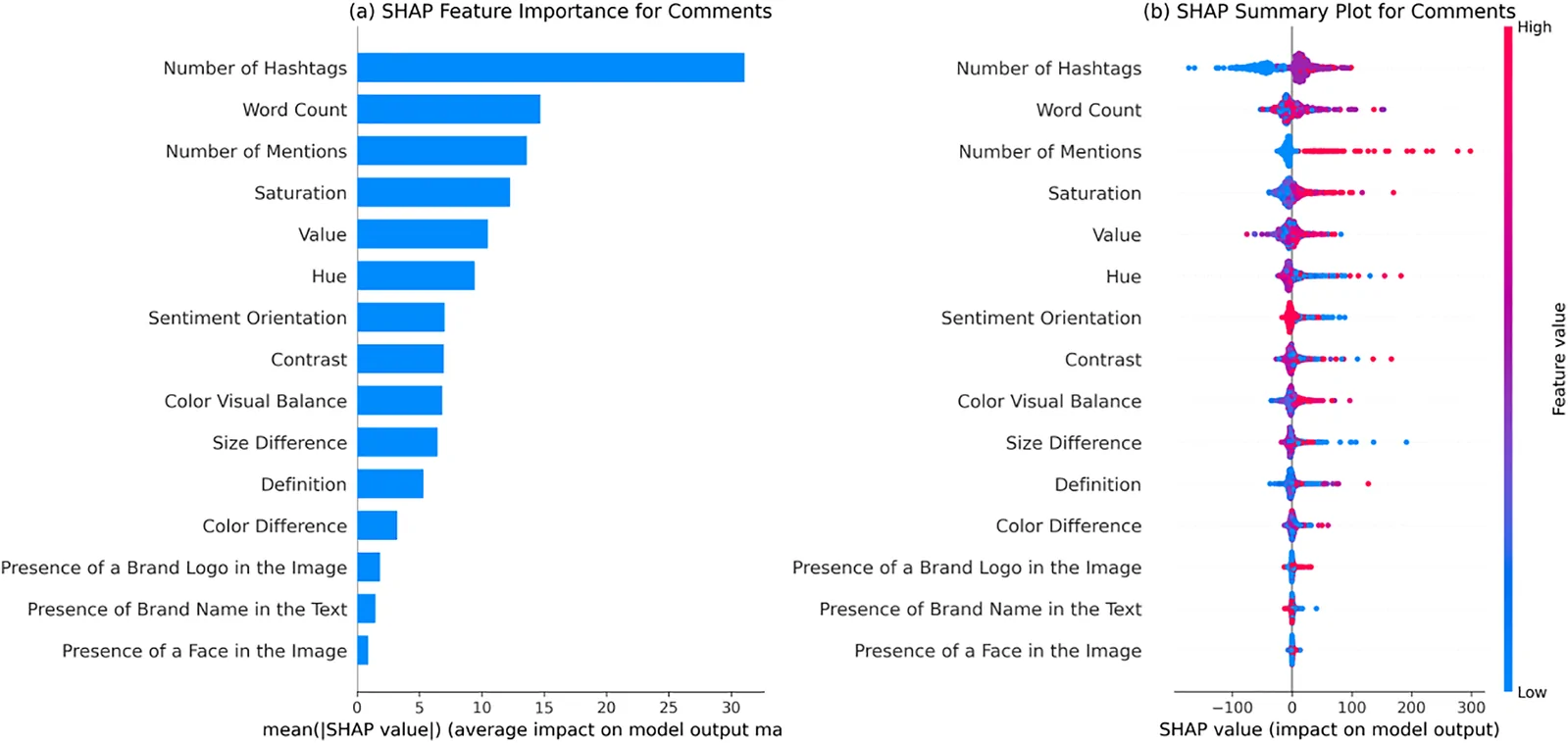
SHAP feature importance for predicting comments.

**Fig 6:**
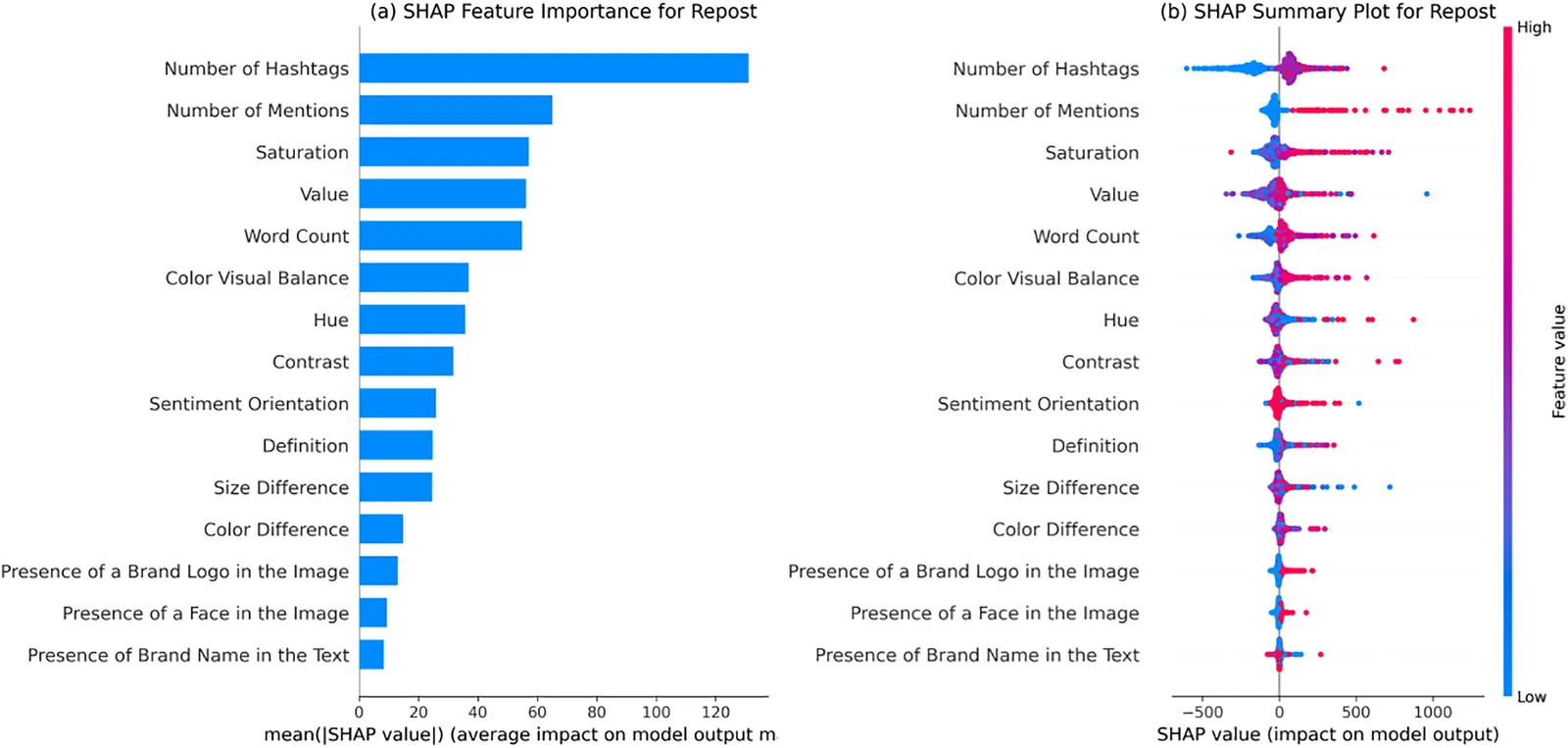
SHAP feature importance for predicting reposts.

**Fig 7:**
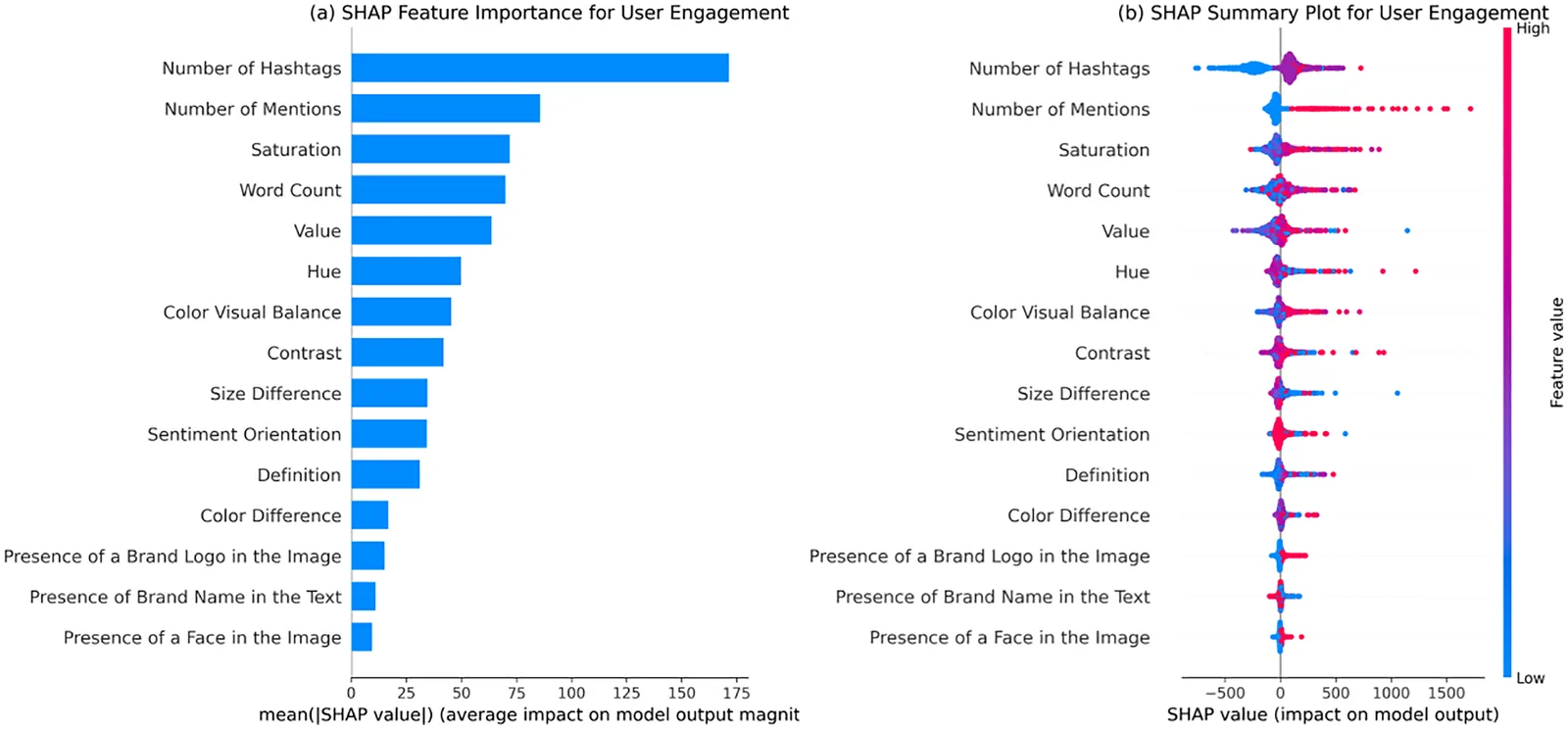
SHAP feature importance for predicting overall engagement.

Building on regression analysis, we use a random forest model to assess the relative importance of each feature in predicting post popularity. As an ensemble method, random forest captures nonlinear relationships and interactions between variables, complementing the regression analysis that highlights individual feature effects. This combination offers a more comprehensive understanding of key factors influencing post popularity. We interpret model outputs using SHAP (SHapley Additive exPlanations), which quantifies each feature’s contribution to the prediction. Figs 4–7 show SHAP-based feature importance for likes, comments, reposts, and overall engagement, respectively, with each figure displaying both mean SHAP values and beeswarm plots for the corresponding engagement type.

For likes, Word Count and Number of Hashtags are the most influential features, suggesting that richer content and effective tagging boost visibility and engagement. Visual features like Hue, brand logos, and Contrast also play significant roles. The beeswarm plot indicates that warmer tones (higher hue) and positive sentiment (red points) are associated with more likes, highlighting the role of emotional tone and visually inviting imagery. Overall, textual characteristics, tagging, and visual attributes are important contributors to the model’s prediction of likes.

For comments, Number of Hashtags stands out, followed by Word Count and Mentions, indicating that tagging, content richness, and social referencing stimulate discussions. Visual features like Saturation, Value, and Hue also matter, while Sentiment Orientation has a moderate effect. The beeswarm plot shows that higher saturation and warmer hues are linked to more comments. Commenting appears more socially driven than liking, with deeper interaction encouraged by tags, references, and emotionally engaging visuals.

For reposts, Number of Hashtags is the top predictor, emphasizing the role of discoverability and sharing. Mentions and Word Count follow in predictive importance, indicating that these features contribute substantially to the model’s prediction of repost volume. Visual features like Saturation, Value, and Hue contribute significantly. The beeswarm plot reveals that vivid colors and higher saturation, especially with warmer hues, are more likely to lead to reposts, indicating the importance of emotional resonance and visual appeal in driving shares.

For overall engagement, Number of Hashtags is the dominant predictor, followed by Mentions, Saturation, Word Count, and Value, highlighting the combined influence of discoverability, interactivity, and content richness. Visual cues like Hue, Color Balance, and Contrast also contribute, while Sentiment Orientation has a moderate effect. The beeswarm plot shows that posts with higher saturation and warmer hues, along with interactive elements like hashtags and mentions, receive stronger engagement, underlining the importance of balanced multimodal content in social media strategy.

## 5 Deep learning-based prediction of post popularity

Building on prior regression analysis identifying key visual and textual features linked to user engagement, our study proposes a predictive framework using multimodal deep learning techniques.

### 5.1 *Model objectives*

The prediction framework has two objectives. The continuous model estimates log-transformed total engagement, while the classification model assigns posts to four popularity levels: low, moderate, high, and viral. Together, the continuous model preserves the full variation in engagement outcomes, while the classification model provides actionable popularity-level categories for content screening.

### 5.2 *Model description*

Both prediction tasks use a shared two-branch multimodal architecture consisting of a VGG19-based image branch and a structured-feature branch.

#### 5.2.1 *Continuous prediction model.*

The continuous prediction model adopts a two-branch architecture. The image branch uses VGG19 pretrained on ImageNet. The original top classification layers are removed, and global average pooling is applied to obtain deep visual representations. The structured-feature branch receives manually extracted image features, text features, posting-time variables, and account-level indicators. These features are standardized and then processed through fully connected layers. The two branches are concatenated, followed by dense layers with L2 regularization and Dropout. The output layer uses a linear activation function to predict log-transformed total engagement.

During model construction, the VGG19 backbone is initialized with ImageNet weights. Its convolutional layers are used for visual feature extraction, and the final layers can be fine-tuned to adapt the visual representation to the Weibo image context. The structured branch includes variables such as color balance, hue, saturation, brightness, clarity, contrast, face presence, logo presence, text length, sentiment, hashtag count, mention count, posting time, days since posting, and follower count.

The outputs from both branches are concatenated and processed through a fully connected network. To prevent overfitting, L2 regularization and Dropout are applied. The final output layer generates a continuous value for post popularity, and the model is trained using the Adam optimizer with mean squared error (MSE) as the loss function.

#### 5.2.2 *Classification prediction model.*

For the classification task, posts are divided into four popularity levels using the 10th, 90th, and 99th percentiles of total engagement. Posts below the 10th percentile are classified as low-popularity posts; posts between the 10th and 90th percentiles are classified as moderate-popularity posts; posts between the 90th and 99th percentiles are classified as high-popularity posts; and posts above the 99th percentile are classified as viral posts. Accordingly, the four popularity levels should be interpreted as categories of aggregate interaction volume rather than balanced profiles of likes, comments, and reposts.

This percentile-based scheme is designed to reflect the long-tailed distribution of social media engagement. The moderate category represents routine engagement outcomes, whereas the high and viral categories distinguish strongly performing posts from the small number of exceptionally popular posts in the upper tail. Thus, the four-level scheme is a decision-oriented operationalization rather than a claim that post popularity naturally consists of four objectively discrete groups.

To reduce dependence on the specific thresholds, sensitivity checks were conducted using alternative cutoffs, including the 5th, 85th, and 99th percentiles and a stricter 99.5th-percentile viral threshold. The results remained qualitatively consistent: macro-F1 changed by less than 0.02, and viral PR-AUC changed by less than 0.05. Detailed results are reported in S1 Appendix.

Importantly, the classification model complements rather than replaces the continuous prediction model. The continuous model retains the full variation in engagement outcomes, whereas the four-level classification provides more interpretable categories for content screening and managerial decision-making.

The classification model follows the same two-branch fusion structure as the continuous model. After the VGG19-based image representations and structured features are combined, the fused representation is processed through fully connected layers with ReLU activation, batch normalization, L2 regularization, and Dropout. The final softmax layer outputs the probability of each popularity class.

### 5.3 *Model training and results*

This section reports the training settings and prediction results for both models, including data partitioning, preprocessing, optimization, regularization, and evaluation metrics.

#### 5.3.1 *Continuous prediction model.*

For the continuous prediction task, the dependent variable was transformed as ln(1+total engagement), where total engagement was calculated as the sum of likes, comments, and reposts. The dataset was randomly divided into training and validation sets using an 80/20 split with a fixed random seed. Structured features were standardized using parameters estimated from the training set and then applied to the validation set. This procedure was used to reduce scale differences among variables and avoid information leakage.

All images were converted to RGB format and resized to 224 × 224 pixels before being input into VGG19. Image augmentation, including random rotation, width-height shifting, and horizontal flipping, was applied only to the training set. The model was trained using the Adam optimizer with a learning rate of 0.0001, mean squared error as the loss function, and mean absolute error as an additional metric. The batch size was set to 32, and early stopping monitored validation loss to restore the best model weights.

On the validation set, the continuous model achieved a log-scale MSE of 1.7421 and MAE of 1.0086. After reversing the log transformation, the MAE on the original engagement scale was 1175.80. These results suggest that the model captures general engagement patterns, but exact count prediction remains difficult because social media engagement is highly skewed and affected by external factors. Therefore, we further developed a categorical prediction model to classify posts into actionable popularity levels.

#### 5.3.2 *Classification prediction model.*

Because the original distribution of the four popularity classes was highly imbalanced, we adopted a class-balanced sampling strategy for the classification task. At each fold, non-overlapping training, validation, and test subsets were constructed. Approximately equal numbers of posts were randomly sampled from each popularity class for model training, preventing the moderate-popularity category from dominating the learning process. A separate class-balanced test set was constructed from observations that did not overlap with the training data, with equal numbers of posts sampled from each class. The validation set consisted of previously unused observations and was used only for early stopping and model selection, rather than for model fitting. This sampling and evaluation procedure was repeated across 10 folds.

Feature standardization was performed using parameters estimated from the training data and then applied unchanged to the validation and test data. This procedure prevented information leakage between the training and evaluation stages.

The classification model was trained using the Adam optimizer and sparse categorical cross-entropy loss. The initial learning rate was set to 0.0005, the batch size was 32, and the maximum number of epochs was set to 100. Early stopping monitored validation loss with a patience of 10 epochs and restored the best weights. A ReduceLROnPlateau scheduler reduced the learning rate by a factor of 0.5 when validation loss did not improve for 5 epochs, with a minimum learning rate of 1e-6.

**Fig 8:**
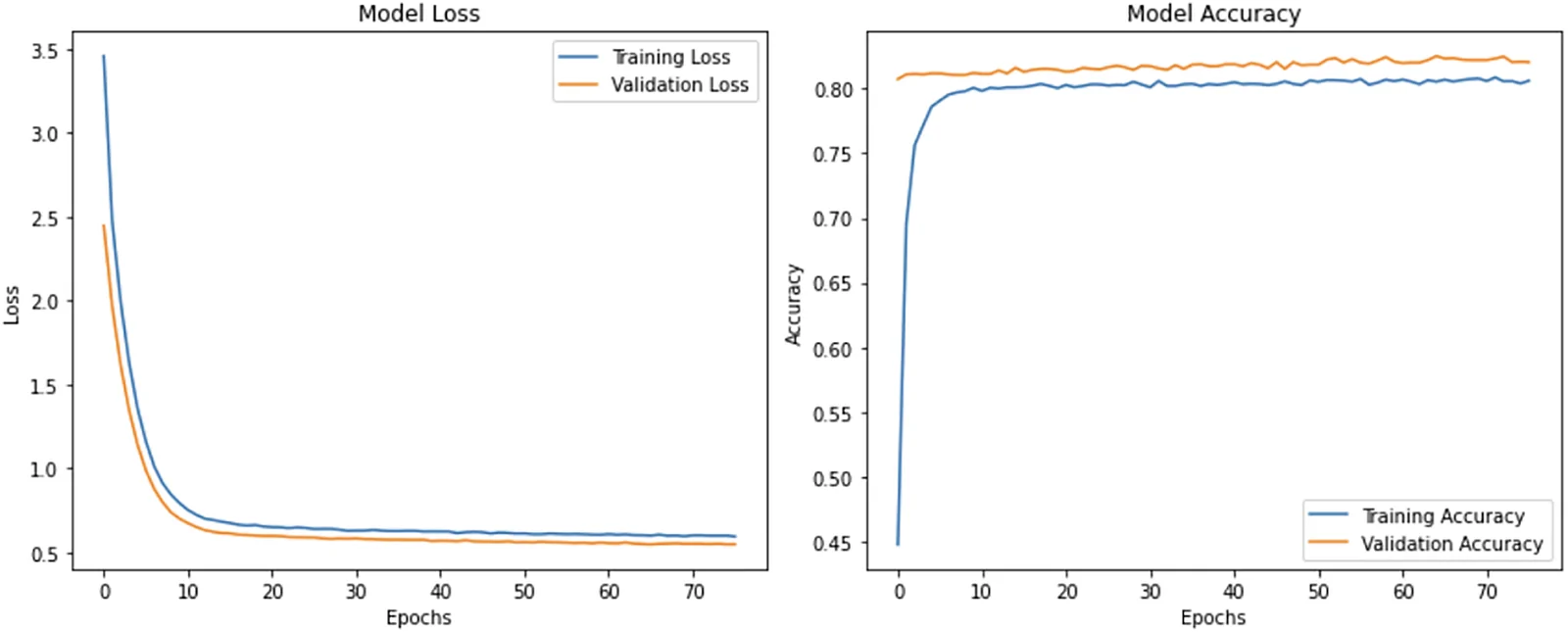
Training and validation performance of the classification prediction model.

Fig 8 reports the training and validation curves for the classification model. The loss and accuracy curves stabilized during training, and the gap between training and validation performance remained limited, suggesting that Dropout, L2 regularization, batch normalization, early stopping, and learning-rate scheduling helped reduce overfitting.

Across the 10 folds, the proposed model achieved an average accuracy of 0.81, macro-precision of 0.79, macro-recall of 0.81, macro-F1-score of 0.80, and one-vs-rest multiclass AUC of 0.80. In practical terms, an average accuracy of 0.81 means that approximately 81% of posts in the class-balanced test folds were assigned to the correct popularity level. The model is therefore intended as a preliminary screening and prioritization tool rather than a deterministic decision rule. Because the four classes were equally represented in the test set, a naive classifier that always predicted a single category would achieve an accuracy of 0.25 rather than 0.80. Therefore, the observed accuracy cannot be attributed to the dominance of the moderate-popularity category. Table 4 further reports precision, recall, and F1-score for each popularity class.

**Table 4:** Class-specific performance of the proposed classification model.

Popularity class	Precision	Recall	F1-score
Low	0.77	0.78	0.77
Moderate	0.86	0.91	0.88
High	0.82	0.84	0.83
Viral	0.71	0.71	0.71
Macro average	0.79	0.81	0.80

As shown in Table 4, the model achieved meaningful precision and recall across all four popularity levels rather than relying primarily on the moderate category. The model performed best for moderate-popularity posts, followed by high-, low-, and viral-popularity posts. The relatively lower performance for viral posts indicates that exceptionally popular content remains more difficult to identify, possibly because the original pool of viral posts is relatively small and their engagement outcomes are more sensitive to external amplification factors.

### 5.4 *Baseline comparison*

To further assess the predictive value of the proposed multimodal deep learning model, we compared it with several baseline classification models, including Logistic Regression, Support Vector Machine, Decision Tree, Random Forest, Gradient Boosting, and Multilayer Perceptron. The baseline models were trained using the extracted structured visual, textual, temporal, and account-level features, whereas the proposed model additionally incorporated VGG19-based deep image representations.

All baseline models and the proposed model used the same class-balanced data partitions, preprocessing procedures, and 10-fold evaluation protocol. Feature standardization parameters were estimated from the training data and then applied unchanged to the validation and test data. Because class imbalance had already been addressed through class-balanced sampling, no additional class weighting was applied to any model. Accuracy, precision, F1-score, and one-vs-rest multiclass AUC were calculated consistently across all models.

As shown in Table 5, the proposed multimodal model achieved the best overall performance. Among the baseline models, the multilayer perceptron achieved the highest accuracy of 0.76, while random forest achieved the highest AUC of 0.75. The proposed model increased accuracy to 0.81 and AUC to 0.80, while also achieving the highest precision.

**Table 5:** Baseline comparison for popularity-level classification.

Model	Accuracy	Precision	F1	AUC
Logistic Regression	0.36	0.72	0.43	0.63
SVM	0.55	0.73	0.61	0.70
Decision Tree	0.71	0.70	0.71	0.58
Random Forest	0.73	0.76	0.75	0.75
Gradient Boosting	0.74	0.74	0.75	0.73
MLP	0.76	0.75	0.75	0.71
**Proposed multimodal model**	**0.81**	**0.79**	**0.80**	**0.80**

Although the absolute improvement is modest, it is meaningful because the baseline models already incorporate structured visual, textual, temporal, and account-level features. The additional improvement therefore indicates that VGG19-based image representations capture latent visual information, such as scene composition, object-level cues, texture, and visual style, that is not fully represented by manually extracted features.

The contribution of the proposed model lies not only in improving predictive accuracy, but also in integrating interpretable structured features with deep visual representations within a unified framework. In practical terms, the improvement can help marketers more accurately screen and prioritize planned posts, particularly when evaluating content at scale. The model also supports both continuous engagement estimation and popularity-level classification, providing more flexible decision support than models based only on manually extracted features.

### 5.5 *Error analysis*

Although the proposed model shows the best overall performance, some prediction errors remain. For the continuous prediction task, the model captures general engagement patterns but has difficulty predicting exact interaction counts, mainly because social media engagement follows a long-tail distribution and is easily affected by external factors such as platform recommendation, campaign timing, trending events, and user diffusion.

For the classification task, errors are more likely to occur between adjacent popularity levels, especially between moderate- and high-popularity posts and between high-popularity and viral posts. This is because these categories are defined by engagement percentiles and their boundaries can be sensitive to small differences in engagement. Therefore, the model is more suitable for identifying popularity potential than for deterministically predicting final engagement outcomes.

## 6 Conclusion and implications

This study examines how multimodal content features are associated with post popularity and evaluates a multimodal framework for continuous engagement estimation and popularity-level classification. The findings provide theoretical implications for content-based popularity assessment and practical guidance for social media content planning.

### 6.1 *Research conclusions*

This study reaches three main conclusions. First, post popularity can be understood as a staged outcome rather than a single engagement count. The four-level framework-low, moderate, high, and viral-captures the long-tail nature of social media engagement and provides a more actionable way to evaluate post popularity.

Second, visual and textual features are associated with post popularity in different ways. Visual attributes such as color balance, saturation, clarity, faces, and logos mainly shape users’ initial attention, perceptual fluency, and visual evaluation, while textual features such as word count, hashtags, mentions, brand-name cues, and sentiment are more closely related to information processing, content discoverability, and interaction intention. These findings suggest that visual features help posts attract attention, whereas textual features play a stronger role in stimulating deeper engagement such as commenting and reposting.

Third, the multimodal prediction model provides a useful tool for identifying popularity potential. By combining VGG19-based image representations with structured content and contextual features, the model performs better than traditional baseline models in popularity-level classification and supports data-driven content decision-making.

### 6.2 *Management implications*

First, the proposed model can be used as a pre-publication screening tool. Before releasing a post, marketers can input the planned image, text, posting time, and account-level information into the model to estimate whether the post is likely to become low-, moderate-, high-, or viral-popularity content. This helps brands identify high-potential posts before publication, rather than relying only on post-hoc evaluation of observed engagement outcomes. Even a moderate improvement in classification performance can improve content prioritization when brands evaluate large numbers of planned posts, helping reduce the risk of allocating promotional resources to relatively low-potential content.

Second, the model can support content optimization before release. If a post is predicted to have low popularity, marketers can revise its visual and textual elements, such as improving image clarity, adjusting color saturation or brightness, strengthening brand cues, refining text length, adding appropriate hashtags, or selecting a more suitable posting time. In this sense, the model provides not only prediction but also feedback for content design. Because the identified feature patterns are associational rather than causal, these recommendations should be treated as diagnostic cues for content development and testing rather than guaranteed optimization rules. Marketers may use the model to generate alternative versions and evaluate them through A/B tests before large-scale release.

Third, the model can assist marketing resource allocation. Posts predicted as high-popularity or viral content can be prioritized for paid promotion, cross-platform distribution, influencer collaboration, or campaign amplification. In contrast, posts predicted as low-popularity content can be revised, delayed, or assigned fewer promotional resources. This allows brands to allocate marketing budgets more efficiently and reduce wasteful promotion.

Finally, the model can be used for campaign evaluation and organizational learning. By comparing predicted popularity with actual engagement outcomes, marketers can identify whether unexpected engagement outcomes are associated with content characteristics or external factors such as platform recommendation, trending events, paid promotion, or campaign timing. This feedback helps brands refine future content strategies and build a more data-driven social media marketing system.

### 6.3 *Research limitations and future directions*

This study provides a framework for post popularity prediction, but several limitations remain. First, our models rely on cumulative engagement outcomes observed at the time of data collection. They do not directly model the temporal growth trajectory of engagement after publication. Future research could use time-series engagement data to examine how posts move across popularity stages, such as from low to moderate, high, or viral popularity.

Second, with the growing diversity of content formats-especially short videos and live streaming-future studies should apply advanced AI and machine learning techniques to extract deep multimodal features and assess how various content formats impact post popularity, offering more targeted marketing strategies.

Third, the model mainly relies on content and observable post-level features and does not directly capture external factors such as platform recommendations, paid promotion, trending events, or network diffusion. Because these factors can substantially affect post exposure, their omission may reduce prediction accuracy, particularly for high- and viral-popularity posts. Therefore, the model should be interpreted as estimating content-based popularity potential rather than deterministically predicting final engagement. Future research could incorporate exposure and platform-level signals to improve prediction accuracy.

Finally, because the study relies on retrospective observational data, the reported associations may be affected by unobserved confounding and should not be interpreted as causal effects. Future research could use controlled experiments, quasi-experimental designs, or longitudinal data to test whether specific content features causally affect engagement.

## Supporting information

S1 Appendix Sensitivity analyses and robustness checks. This file includes sensitivity analyses for alternative popularity-tier cutoff schemes, heteroskedasticity and multicollinearity diagnostics, alternative count-model specifications, and quantile regression results.
(DOCX)
