# Supplementary material for: Predicting social media post popularity using multimodal deep learning: Insights from content features
Source: PLoS One. 2026 Aug 26;21(8):e0356612. doi: 10.1371/journal.pone.0356612 (PMC13513989; doi:10.1371/journal.pone.0356612)
Supplement: Supplementary file 1 [file pone.0356612.s001.docx]

**S1 Appendix. Sensitivity analyses and robustness checks**

**Appendix A**. Heteroskedasticity and Multicollinearity Diagnostics

**A1. Breusch–Pagan tests for heteroskedasticity**

Heteroskedasticity is present across OLS specifications; hence HC3-robust standard errors are reported in the main text.

| **Model** | **BP LM stat** | **BP LM p-value** | **BP F stat** | **BP F p-value** |
| --- | --- | --- | --- | --- |
| Likes (log) | 728.605 | 0.000 | 32.711 | <0.001 |
| Reposts (log) | 1,450.185 | 0.000 | 70.729 | <0.001 |
| Comments (log) | 1,078.777 | 0.000 | 50.376 | <0.001 |
| Total engagement (log) | 1,232.501 | 0.000 | 58.586 | <0.001 |

Note: BP = Breusch–Pagan; p-values rounded to three decimals.

**A2. Variance Inflation Factors (VIF)**

**Before mitigation.** Two subject–background separation metrics exhibit high collinearity:

Color Difference VIF = 25.94

Size Difference VIF = 14.76

Next highest among other predictors: value VIF = 4.96; remaining VIFs ≲ ~2.8

**Mitigation.** We construct a standardized composite index

SB_index = z(Size Difference) + z(Color Difference), replacing the two original variables in robustness checks.

After mitigation (with SB_index). All VIFs fall well below conventional thresholds (max < 3):

| **Predictor (Top entries post-mitigation)** | **VIF** |
| --- | --- |
| text_contain_logo | 2.80 |
| hashtag_num | 2.75 |
| sentiment_prob | 2.08 |
| face | 1.80 |
| text_num | 1.78 |
| contrast | 1.66 |
| logo | 1.57 |
| value | 1.48 |
| visual_balance_color | 1.42 |
| SB_index | **1.30** |
| (others) | ≤ 1.30 |

Summary: After aggregation, no severe multicollinearity remains (all VIF < 3).

**Appendix B.** Alternative Specifications and Tail Behavior

**B1. Poisson vs. Negative Binomial (count outcomes)**

Likelihood ratio tests strongly favor Negative Binomial (NB) over Poisson for raw counts.

| **Outcome** | **LR stat (NB vs Poisson)** | **p-value** |
| --- | --- | --- |
| Likes | 487,875.175 | <0.001 |
| Reposts | 6,817,160.745 | <0.001 |
| Comments | 1,380,248.174 | <0.001 |

Note: p≈0 for all three outcomes; NB is preferred.

**B2. Negative Binomial IRRs (NB2)**

Illustrative results for Likes (full IRR tables for Likes/Reposts/Comments in Table C1, Supplementary):

| **Outcome** | **Predictor** | **IRR** | **95% CI (lower, upper)** | **p-value** |
| --- | --- | --- | --- | --- |
| Likes | visual_balance_color | 0.968 | (0.944, 0.994) | 0.015 |
| Likes | size difference | 0.837 | (0.771, 0.909) | <0.001 |
| Likes | color difference | 0.771 | (0.691, 0.860) | <0.001 |
| Likes | hue | 1.003 | (0.981, 1.024) | 0.817 |

Interpretation: For example, a one-unit increase in visual_balance_color is associated with a 3.2% decrease in expected Likes (IRR=0.968). Directions match OLS (log-scale) results.

**B3. GLM–Negative Binomial (stability check)**

A generalized linear model with NB family yields consistent IRRs and converges without warnings.

| **Outcome** | **Predictor** | **IRR** | **SE** | **p-value** |
| --- | --- | --- | --- | --- |
| Likes (GLM-NB) | visual_balance_color | 0.968 | 0.0123 | 0.009 |
| Likes (GLM-NB) | size difference | 0.837 | 0.0396 | <0.001 |
| Likes (GLM-NB) | color difference | 0.771 | 0.0524 | <0.001 |
| Likes (GLM-NB) | hue | 1.002 | 0.0106 | 0.817 |

**B4. Quantile Regression (τ = 0.25, 0.50, 0.75, 0.90) for log(Total Engagement)**

Quantile regressions preserve the signs and inferences of key predictors across the distribution, with some tail-specific nuances. Sign-consistency summary (Main OLS vs. QR signs):

| **Predictor** | **Main OLS** | **τ=0.25** | **τ=0.50** | **τ=0.75** | **τ=0.90** |
| --- | --- | --- | --- | --- | --- |
| visual_balance_color | – | – | – | – | – |
| size difference | + | + | + | + | + |
| color difference | + | + | + | + | + |
| hue | + | – | + | + | + |
| saturation | + | + | + | + | + |
| value (V) | – | – | – | – | + |
| definition (clarity) | + | – | – | + | + |
| contrast | – | – | – | – | – |
| face | – | – | – | + | + |
| logo | + | + | + | – | – |
| text_num | + | + | + | + | + |
| hashtag_num | + | + | + | + | + |
| at_num | + | – | – | + | + |
| text_contain_logo | + | + | + | + | + |
| sentiment_prob | – | – | – | – | + |

Notes:

(1) Positive effects of saturation, hashtags, and text length strengthen toward the upper tail (τ≥0.75).
(2) Value (V) turns positive at τ=0.90, indicating very bright images may help only among high-engagement posts.

**Appendix C.** Sensitivity of Popularity-Tier Definitions to Percentile Cutoffs (10-fold Cross-Validation; Mean ± SD)

| Scheme | Percentile cutoffs | Class share (L/M/H/V) | Overall Acc | Macro-F1 | **Viral F1** | **Viral PR-AUC** | Note |
| --- | --- | --- | --- | --- | --- | --- | --- |
| **Baseline** | ≤10th, 10th–90th, 90th–99th, ≥99th | 10% / 80% / 9% / 1% | **0.81 ± 0.01** | **0.76 ± 0.01** | **0.41 ± 0.03** | **0.58 ± 0.03** | Main specification |
| **Alt-1** | ≤5th, 5th–85th, 85th–99th, ≥99th | 5% / 80% / 14% / 1% | 0.80 ± 0.01 | 0.76 ± 0.01 | 0.42 ± 0.03 | 0.59 ± 0.03 | More High-tier samples; overall stable |
| **Alt-2** | ≤10th, 10th–95th, 95th–99.5th, ≥99.5th | 10% / 85% / 4.5% / 0.5% | 0.82 ± 0.01 | 0.75 ± 0.01 | 0.34 ± 0.04 | 0.54 ± 0.04 | Viral class rarer; F1 declines |

Note: Values are reported as mean ± SD across 10 folds. Alt-2 uses a stricter viral threshold, which reduces the number of viral posts and therefore lowers Viral F1.
